# Validating a digital depression prevention program for adolescents in Jordan: cultural adaptation and user testing in a randomized controlled trial

**DOI:** 10.3389/fpsyt.2025.1529006

**Published:** 2025-02-12

**Authors:** Latefa Ali Dardas, Obada Al-leimon, Tracy Gladstone, Abd Arrahman Dabbas, Insaf Alammouri, Benjamin Van Voorhees

**Affiliations:** ^1^ Department of Community Health Nursing, School of Nursing, The University of Jordan, Amman, Jordan; ^2^ School of Medicine, The University of Jordan, Amman, Jordan; ^3^ Hassenfeld Child Health Innovation Institute, Department of Behavioral and Social Sciences, School of Public Health, Brown University, Providence, RI, United States; ^4^ Department of Pediatrics, University of Illinois at Chicago, IL, United States

**Keywords:** digital health intervention, adolescent mental health, depression prevention, cognitive behavioral therapy (CBT), cultural adaptation

## Abstract

**Purpose:**

Digital health interventions (DHIs) offer scalable solutions for improving mental health care access in underserved settings. This study is part of a multi-phased project aimed at adapting a depression prevention DHI for Jordanian adolescents. It evaluated the feasibility, cultural acceptability, and effectiveness of the translated and culturally adapted DHI, named Al-Khaizuran, with comparisons to school-based group CBT.

**Methods:**

A two-arm, single-blind randomized controlled trial with a mixed-methods design was conducted among 109 Jordanian adolescents aged 15–17 years experiencing mild to moderate depression. Participants were randomly assigned to either Al-Khaizuran DHI (n=55) or school-based group CBT (n=54). The adaptation of Al-Khaizuran DHI components was guided by the Ecological Validity Framework, while the procedural adaptation followed Barrera and Castro’s Heuristic Framework, incorporating iterative refinement based on user feedback and contextual considerations.

**Results:**

Al-Khaizuran DHI was found to be a culturally relevant and acceptable intervention for Jordanian adolescent. Over half of the participants reported that the intervention was effective, empowering, and easy to use, with 51% expressing satisfaction and willingness to recommend it. However, challenges such as limited access to personal devices, privacy concerns, and participants’ reliance on shared family resources emerged as significant barriers to consistent engagement. Participants showed a preference for individualized, blended interventions, with a significant reduction in support for group CBT. No significant difference was found in depression scores between the two groups. However, the Al-Khaizuran DHI group demonstrated higher post-intervention resilience scores (p=0.026). Beliefs about the effectiveness of the intervention significantly predicted behavioral intention (p=0.022), while perceived difficulty was a barrier to adherence (p=0.015).

**Conclusions:**

Al-Khaizuran DHI exemplifies the potential of culturally adapted digital interventions in bridging mental health care gaps in resource-limited settings. However, its effectiveness is contingent upon addressing barriers to access, enhancing program interactivity, and integrating hybrid support systems that combine digital tools with in-person guidance. Future implementations should consider strategies to actively engage parents to foster a supportive environment that promotes the well-being of adolescents.

**Clinical trial registration:**

https://doi.org/10.1186/ISRCTN14751844, identifier ISRCTN14751844.

## Introduction

The Arab region, consisting of 22 countries and a population of approximately 425 million, has one of the largest youth demographics globally, with 60% of its population under the age of 25 (4). Mental health disorders are a critical concern in this region (1), with their burden, measured by disability-adjusted life years (DALYs), exceeding the global average in many Arab countries (2). Arab Adolescents are particularly at risk due to their heightened exposure to social, political, and economic stressors (1, 3). Among this demographic, depression is projected to be the most prevalent mental health disorder (5, 6).

Jordan, as part of the Arab region, exemplifies this challenge, with a national study reporting a 34% prevalence rate of moderate to severe depression among adolescents aged 12 to 17 years. Of these, 19% experience moderate symptoms, and 15% face severe symptoms (7). Depression severity was notably higher among female adolescents aged 14–17 years, those from families with lower monthly incomes, and individuals with chronic medical or mental health conditions (7). Despite the evident need for mental health interventions, Jordan, like many Arab countries, faces a critical shortage of mental health resources. Screening, treatment, and prevention practices for depression remain far below recommended standards. Mental health services are primarily limited to two psychiatric hospitals, a few psychiatric units in general hospitals, and outpatient clinics, where the focus is predominantly on psychopharmacological treatments. Psychotherapy services are underdeveloped, with minimal availability for families of individuals with depression (3). Furthermore, while Jordan has committed to integrating mental health services into its primary health care system, progress toward implementation has been minimal (1, 3). These systemic challenges are further exacerbated by a significant shortage of trained mental health professionals, including psychiatrists, psychologists, and psychiatric nurses, as well as pervasive stigma surrounding mental health issues, which discourages individuals from seeking care (8–12). Addressing these barriers necessitates scalable, culturally appropriate, and innovative strategies to enhance mental health care access and outcomes.

According to the World Health Organization (WHO) (13), addressing the barriers faced by underserved populations is essential for improving global health equity. These barriers can be mitigated through low-intensity interventions, which are designed to be resource-efficient and accessible to a large number of individuals. Examples include self-help programs and interventions delivered by non-professionals (14–17). Among such approaches, digital health interventions (DHIs) have emerged as promising tools due to their ability to provide anonymity, flexibility in time and location, accessibility, and scalability. These characteristics make DHIs particularly suited to overcoming structural barriers to healthcare access (18–21). Evidence has demonstrated the effectiveness of DHIs in preventing and treating mental health disorders (22–26). However, most DHIs have been developed and tested primarily in high-income countries for majority populations. Their effectiveness is often reduced when applied to individuals from diverse cultural or ethnic backgrounds, who face additional barriers such as language, cultural differences in understanding disease and treatment, or limited knowledge of the healthcare system (17, 27–31). These challenges highlight the importance of incorporating cultural considerations into intervention development to address the unique needs of different populations (32). While creating entirely new interventions for each cultural context requires substantial resources, an alternative approach is to adapt existing evidence-based psychological treatments to align with the cultural context of the target population (33).

CATCH-IT (Competent Adulthood Transition with Cognitive Humanistic and Interpersonal Training) is an internet-based depression prevention program designed to help adolescents build resilience and prevent depression. It integrates components of behavioral activation, cognitive behavioral therapy (CBT), and interpersonal therapy. The program was developed to target adolescents with sub-threshold depressive symptoms and provide an acceptable, low-cost, and broadly available intervention/prevention model of depressive disorders (34). In addition, its techniques have been shown to impact a range of emotional and psychological issues, including anxiety, self-harm risk, and emotional resilience (35, 36). The program is based on the understanding that a large majority of individuals experience sub-threshold depressive symptoms, which do not currently meet the criteria for major depressive disorder but often progress to it over time (37, 38). Sub-threshold depressive symptoms, such as minor depression (defined as two symptoms persisting for more than one week), are associated with significant costs and impairments in social and academic functioning (39, 40). Consequently, early or preventive interventions targeting individuals with sub-threshold symptoms have been consistently recommended to reduce the overall burden of depressive disorders (41, 42).

Early studies of the program have demonstrated its effectiveness in reducing depressive symptoms, with improvements observed over multiple timeframes, including 6, 12, and 30 months (43–45). For example, Van Voorhees et al. conducted a randomized clinical trial within primary care settings and found that adolescents who participated in CATCH-IT had significantly reduced depressive symptoms over the 12-week intervention period (43). This initial success was further supported by longer-term studies, such as Saulsberry et al. (45), which reported positive outcomes at the one-year mark for preventing adolescent depression in primary care settings, and Richards et al. (44), who found sustained improvements in depressive symptoms up to 2.5 years post-intervention. These findings were corroborated by Gladstone et al., who compared CATCH-IT to an internet-based general health education program. They demonstrated that CATCH-IT participants with elevated symptoms of depression at baseline reported fewer episodes of depression at follow-up, reinforcing its effectiveness in addressing mental health concerns among adolescents (46). Another study found that adolescents with sub-threshold depressed mood showed a clear preference for innovative behavioral treatment approaches, such as CATCH-IT, for depression prevention (47). International studies have also expanded the reach of CATCH-IT, supporting its utility across diverse cultural contexts. For instance, a study by Sobowale et al. (48) adapted the program for Chinese adolescents, and it was later piloted, showing that the culturally adapted version significantly reduced depression and anxiety symptoms among this group (49).

Despite its promise and public health relevance, CATCH-IT and similar DHIs have not been tested in the Arab region, leaving a critical gap in understanding their effectiveness within this cultural context. A study by Al Dweik et al. (50) systematically reviewed DHIs for mental health in the Arab region, examining their opportunities and challenges. The study highlighted the diverse modalities and platforms used to address various mental health conditions and emphasized the potential of digital tools to enhance access to care and reduce stigma. However, it also underscored significant gaps, such as limited cultural tailoring of interventions, lack of integration with broader mental health systems, and insufficient evidence on long-term outcomes. The findings point to the urgent need for more culturally sensitive, scalable, and comprehensive DHIs to address mental health challenges effectively in the region.

These recommendations are particularly relevant given the remarkable growth in internet penetration across the Arab region over the past decade, driven by advancements in digital infrastructure, increased smartphone affordability, and widespread adoption of social media platforms (51, 52). As of 2023, internet engagement in the Arab region slightly exceeded the global average internet penetration rate of approximately 64.5% (51, 52). In Jordan specifically, adolescents rank among the highest internet users in the region (53), with activities such as social media, online gaming, and instant messaging forming integral parts of their daily lives. A recent report indicated that 97.1% of males and 95.8% of females aged 15–19 have internet access in Jordan (54).

## The current study

The widespread of internet usage among adolescents in Jordan presents a significant opportunity to implement DHIs targeting mental health issues such as depression. Therefore, this research thrived in its mission to explore the potential of culturally adapted digital interventions for addressing mental health challenges among Jordanian adolescents. With a focus on scalability and cultural alignment, this initiative represents a pioneering effort to provide innovative and accessible solutions for underserved youth populations in the country. This research is grounded in findings from meta-analyses that highlight the importance of cultural adaptation, suggesting that treatments tailored to populations for whom the intervention was not originally designed are significantly more effective than non-adapted versions (55, 56).

This research has also addressed factors that need consideration when introducing new interventions, including the motivational and behavioral predictors that influence adolescents’ engagement. Research has shown that organizing factors according to the Theory of Planned Behavior framework can effectively predict adolescents’ and young adults’ perceived need for treatment and their intention to accept a physician’s diagnosis of major depression (57, 58). This theory offers a comprehensive framework for understanding the factors that drive motivation, intention, and adherence to behaviors. In this model, intention is considered the most immediate precursor to behavior and is influenced by three key components: attitudes and beliefs regarding the behavior (e.g., attitudes toward an intervention), subjective norms (e.g., the influence of family, peers, or employers), and perceived behavioral control (e.g., self-efficacy, or the belief in one’s ability to perform the behavior) (59). For example, in the context of adhering to a depression intervention, intention is shaped by how individuals perceive the effectiveness and importance of the intervention, the social pressures they feel from their environment, and their confidence in their ability to follow through. Understanding the attitudinal predictors of motivation and adherence is crucial for mental health care providers, health policy planners, and prevention researchers, particularly when designing interventions that seek to enhance motivation and adherence to preventive care or digital health interventions.

### Study aims and hypotheses

Primary aim 1: Evaluate the feasibility and cultural acceptability of Al-Khaizuran DHI among Jordanian adolescents, and compare its acceptability to targeted school-based group CBT. We hypothesize that Al-Khaizuran DHI will be feasible, culturally acceptable, and demonstrate higher engagement and more favorable acceptability ratings than school-based group CBT

Primary aim 2: Evaluate the comparative effectiveness of Al-Khaizuran and school-based group CBT in preventing the onset of depressive episodes and improving other patient-centered outcomes (depressive symptoms, resiliency, and intervention attitudes and preferences) among Jordanian adolescents. We hypothesize that both Al-Khaizuran and school-based group CBT will be comparably effective in preventing depressive episodes and improving other patient-centered outcomes.

Secondary aim 1: To explore the role of adolescents’ attitudes and beliefs regarding the intervention’s effectiveness as predictors of their intention to adopt preventive behaviors and engage in behavioral changes. We hypothesize that these attitudes and beliefs will be strong predictors of their intention to make behavioral changes and engage in preventive strategies.

## Guiding frameworks

Two frameworks were employed to guide the content and procedural components of the cultural adaptation of the CATCH-IT program for Jordanian adolescents. The first was the *Ecological Validity Framework* by Bernal et al. (60), which informed the adaptation of intervention components across eight key domains: language (translation and regional or subcultural differences), persons (roles and patient–therapist relationships), metaphors (symbols and sayings), content (values, customs, and traditions), concepts (theoretical model of the treatment), goals (alignment between therapist and patient objectives), methods (procedures for achieving treatment goals), and context (broader social, economic, and political environments). The second framework, *Barrera and Castro’s Heuristic Framework for the Cultural Adaptation of Interventions* (61), guided procedural adaptations. This model outlines four steps: (1) gathering information through literature reviews or qualitative research, (2) developing a preliminary adaptation based on the information collected, (3) testing the preliminary adaptation through pilot or case studies, and (4) refining the adaptation based on findings from these studies. Below is a detailed description of how the two frameworks were used to guide the study.

## The Al-Khaizuran project: a phased approach to developing a culturally adapted digital intervention for Jordanian adolescents

Al-Khaizuran, meaning “Bamboo” in English, was chosen as the adaptation project’s name to symbolize the remarkable resilience of bamboo, a plant known for its rapid growth, ability to thrive in diverse environments, and flexibility that allows it to bend without breaking even in strong winds or heavy rains.

### Procedural adaptation (phases 1-4, 6-7)

**Figure 1:**
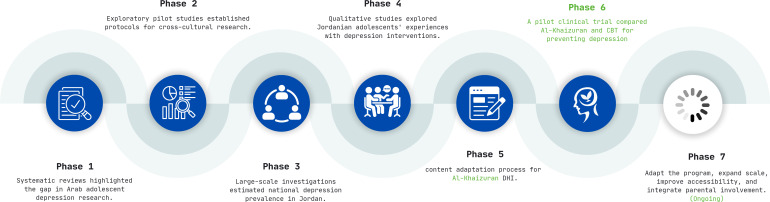
Al-Khaizuran DHI development phases.

Based on the aforementioned theoretical frameworks, a systematic and comprehensive scientific approach was adopted to ensure that the intervention was evidence-based, culturally relevant, and tailored to the specific needs of the target population. This process followed seven distinct phases for intervention development. Phase one included systematic reviews that explored the mental health profile of Arab adolescents, particularly depression. This work highlighted a significant gap in the literature in terms of availability of prevalence data and culturally competent tools and interventions, which made it difficult to design, implement, and disseminate effective programs to restore, maintain and promote the mental health of Arab adolescents (3, 10). Building on these results, phase two included a series of exploratory pilot studies establishing an evidence-based protocol for conducting cross-cultural research in Jordan, as an Arabic country exemplar, taking into consideration all ethical, methodological, cultural, linguistic, and logistical issues that might potentially affect the validity and reliability of collected data (62, 63). In phase three, the work moved to large-scale investigation that aimed at estimating the national prevalence of depression among adolescents in Jordan (7). This work also identified characteristics associated with the prevalence and severity of these problems, including sociodemographic and health characteristics (8, 64, 65). National data were also utilized to compare and contrast available depression assessment tools often utilized in the Arab context and introduce the most appropriate factor structure for this population (66). Phase four encompassed qualitative studies centered on Jordanian adolescents suffering from depression, aiming to capture their experiences and dysfunctional attitudes toward various depression interventions. During these studies, the translated and adapted concept and methodology were presented to the participants. Their comprehensive feedback, including both positive and negative aspects, was meticulously analyzed and incorporated to enhance and develop the intervention strategies (67, 68). Phase 5 included the content adaptation process detailed in the next section. Phase six, which is presented in this paper, includes a mixed methods randomized clinical trial described in details in next sections. Phase seven of the project is currently underway, involving modifications based on phase 6. During this phase, we are refining the program based on the insights gathered and preparing for its broader implementation (**Figure 1**).

### Content adaptation (phase 5)

The content adaptation process for Al-Khaizuran focused on six key aspects to ensure cultural relevance and usability for Jordanian adolescents. These aspects included characters, activities, language translation, concepts of mental health and treatment, structure, and functionality, each adapted to align with the cultural and contextual needs of the target population (32, 69). See **Table 1**.

**Table 1:** The content adaptation process for Al-Khaizuran.

Aspect	Specific Components	Examples
Characters	Appearances/Names	Changing "Lindsay" to "Laila" or "Ahmad"
	Content/Stories/Background	Adding a character navigating social pressures in Jordan
	Role of Characters	Replacing authoritative narrators with friendly mentors
Activities	Daily Life	Including communal activities like family dinners
	Gender-Specific Behavior	Boys playing soccer, girls engaging in artistic workshops
	Religious/Traditional Activities	Incorporating prayers or Ramadan-related practices
	Coping Strategies	Using family support as a stress-relief example
Language Translation	Simplification	Replacing complex terms with straightforward, relatable phrases
	Cultural Language Nuances	Adapting idiomatic expressions for local context
Concepts	Religious Teachings	Sharing Quranic verses emphasizing the importance of avoiding stigmatizing behaviors and promoting acceptance of psychological problems.
	Guidance from Specialists	Promoting the Quranic instruction to encourage seeking advice from mental health professionals.
	Stigma Reduction	Framing depression prevention as academic performance support
Structure	Simplification of Modules	Reducing 14 modules to 10 concise sessions
	Storytelling	Using engaging stories to convey therapeutic concepts
Functionality	Mobile-Friendly Design	Optimizing the app for mobile devices
	Enhanced Navigation	Adding flowcharts and visual guides for usability

The first aspect, characters, involved significant changes to ensure relatability. The names of the characters were adapted to resonate with local culture, replacing Western names such as “Lindsay” with culturally relevant names like “Laila” or “Ahmad.” Additionally, new character backgrounds were introduced, such as a young girl navigating social pressures in Jordan, reflecting the lived experiences of adolescents in the region. The roles of narrators were also modified, shifting from authoritative figures to more conversational and approachable styles, akin to a friendly elder or mentor sharing advice, rather than an expert lecturing.

Activities were another focal point of adaptation. Daily life activities were redesigned to prioritize communal and family-oriented practices, such as group games or community events, over solitary tasks. Gender-separated activities were included, such as boys playing soccer and girls engaging in artistic workshops, to align with societal norms. Exercises incorporating religious and traditional practices, like prayer or storytelling around religious occasions, were added to enhance cultural relevance. Moreover, coping strategies were tailored to local contexts, incorporating examples like seeking guidance from a trusted elder or connecting with extended family for support.

For language translation, the text was simplified to ensure easier comprehension, removing overly technical terms and adapting to the reading level of adolescents. Cultural language nuances were also addressed by replacing metaphors and idiomatic expressions with phrases more familiar to the Jordanian audience, ensuring the intervention felt accessible and relatable.

The concepts of mental health and treatment were framed in culturally sensitive ways. Mental health was often explained through regular symptoms like fatigue or lack of energy, which are commonly recognized expressions of distress in the region. To reduce stigma, the intervention positioned depression prevention as a tool for stress management or academic performance enhancement, making the subject matter more approachable and less stigmatized. The Islamic religious context of participants was utilized as an enabler. Islamic teachings emphasize the importance of seeking treatment and discourage harmful labeling or mocking of others. For instance, a verse from the Holy Quran states: *“Let not some men among you laugh at others, it may be that the latter are better than the former. Nor let some women laugh at others, it may be that the latter are better than the former. Nor defame nor be sarcastic to each other, nor call each other by offensive nicknames … And those who do not desist are indeed doing wrong.”* (Al-Hujuraat: 11). This verse highlights the prohibition of behaviors that foster hatred or disrupt societal harmony, stressing that actions like mocking or name-calling are sinful and require sincere repentance (Taubah). These principles extend to interactions with individuals experiencing mental illnesses, as Islam categorically rejects stigmatizing beliefs, attitudes, and behaviors. Additionally, another verse advises: *“If ye realize this not, ask of those who possess the Message.”* (Al-Nahl: 43). This underscores the importance of consulting specialists in fields where one lacks knowledge, including mental health, rather than relying on unscientific or traditional approaches. Integrating these aspects into mental health interventions has been found promising (10).

The program’s structure underwent significant adjustments. The original 14 modules were condensed into 10 shorter, focused sessions to maintain participant engagement while retaining the therapeutic value of the content. Dense academic descriptions were replaced with engaging storytelling to make the material more relatable and appealing for young participants.

Finally, functionality was adapted to align with the technological habits of the target population. Recognizing the widespread use of smartphones among Jordanian adolescents, the program was optimized for mobile accessibility rather than a website. Navigation was enhanced with visual aids, including tables and flowcharts, to simplify the user experience and ensure the app was easy to use. These adaptations collectively aimed at producing Al-Khaizuran as both culturally relevant and user-friendly, increasing its potential to effectively support mental health among Jordanian adolescents. Fidelity checks were conducted throughout the adaptation process to ensure that the main therapeutic components of CATCH-IT, including behavioral activation, CBT, and interpersonal therapy, were preserved. These checks involved regular reviews by clinical experts to ensure that the adapted modules remained aligned with the evidence-based foundations of CATCH-IT, while also being culturally appropriate for Jordanian adolescents.

## Mixed methodology

### RCT design

A two-arm, single-blind, individually randomized treatment trial was conducted. Participants were assigned to either Al-Khaizuran DHI or school-based group CBT. Outcomes for clinical effectiveness were assessed at baseline and postintervention. This study was prospectively registered on the ISRCTN registry (ISRCTN14751844) and received local ethical approval from the University of Jordan (number: 19.2018.1106) and the Jordanian Ministry of Education (number: 3.10.22420). The study was conducted in the two largest governorates in Jordan, Amman and Zarqa, and involved two large public schools that enroll students from a wide range of socioeconomic backgrounds. All participating students were already enrolled in these schools with the intervention designed to integrate seamlessly within the school environment, ensuring accessibility and ease of participation for all students.

### Qualitative interviews design

To support or clarify the quantitative results gathered by the study questionnaire, we conducted a series of semi-structured phone interviews with eight participants, consisting of an equal number of males and females. Each interview lasted approximately 20 minutes and aimed to explore the students’ experiences and perceptions of the program. The interviews were designed to capture in-depth insights into how the program influenced the participants, focusing on their engagement, the perceived benefits, and any challenges they encountered during their participation. To ensure a diverse range of perspectives on the intervention, participants were selected based on their engagement with the program, categorized into three groups according to the percentage of modules they completed: High engagement: Participants who completed 75–100% of the modules (n=3); Moderate engagement: Participants who completed 40–74% of the modules (n=3); and Low engagement: Participants who completed 0–39% of the modules (n=2).

The qualitative interview guide for evaluating the website is structured into four key sections: (1) Usage and Engagement: explores interaction patterns, with questions like, “How often do you visit the website?” and “What features or sections of the website do you use most frequently?”, (2) Effectiveness and Impact: assesses the site’s influence on mental health, with questions such as, “Has using the website helped you manage or reduce symptoms of depression?” and “Can you provide specific examples of how the website has been beneficial to you?” (3) The Usability and Design: focuses on ease of navigation and user interface, asking questions like, “How easy is it to navigate the website?” and “Have you encountered any technical issues or bugs while using the website?”, and (4) Suggestions for Improvement: invites feedback for enhancements, with prompts like, “What do you like least about the website?” and “What would it be if you could change one thing about the website?”.

## Inclusion criteria

Eligible participants consisted of male and female school adolescents aged 13-17 years who exhibited mild to moderate depression (scores ranging between 35 and 49 on the Beck Depression Inventory-II). Individuals with severe depression scores were not included in the study. This is primarily because CATCH-IT program is designed as a preventive intervention aimed at adolescents experiencing subthreshold depressive symptoms, not those with severe symptoms or current major depressive disorder. Additionally, adolescents with comorbid psychiatric conditions, such as bipolar disorder, thought disorders, conduct disorders, or substance use disorders, were excluded since these conditions require different or additional treatments. Adolescents presenting with severe suicidality at baseline (based on the Suicide Intent Scale and the Suicide Ideation Scale) were also excluded. Instead, specific procedures were implemented to refer these cases for urgent care, as detailed in the ethical considerations section. Furthermore, adolescents already receiving other forms of depression treatment outside of the study were excluded to prevent contamination of the study results.

## Power analysis

The required sample size for this study was calculated based on a statistical test of difference between two independent means (groups). The effect size was estimated as medium based on previous evaluations of the CATCH-IT program (35, 36, 44–46, 49, 69). Using a significance level of 0.05, with a power level of 0.8, a minimum sample size of 106 participants was required. A retrospective power analysis was conducted to determine if the study had sufficient power to detect meaningful differences between the intervention groups. The observed mean difference between groups, the pooled standard deviation, the sample sizes of each group, and the significance level (α=0.05) were used to calculate the statistical power of the study. The power calculation was then performed using a two-sample t-test for independent groups, assuming a two-tailed test. Results indicated that the sample size provided sufficient power to detect medium to large effect sizes, which aligns with the anticipated impact based on prior evaluations of similar interventions. However, the power to detect smaller effect sizes may have been limited due to the sample size constraints of this study.

## Recruitment and consenting

Parents were invited to participate through SMS messages sent by the school principals, as this is the customary method of communication between school administrators and parents. Participation was voluntary, and parents who agreed to participate responded to the invitation by confirming their willingness. Boys and girls whose parents consented to their participation received a comprehensive package containing all study details. This package provided clear and thorough information about the study’s objectives, procedures, and expectations to ensure informed participation. Those who agreed to participate were invited to a meeting with the study Principal Investigator. During this meeting, the procedures were explained in detail once again, ensuring that both participants and their parents fully understood the study. Any questions or concerns were addressed comprehensively. After this thorough discussion, written assents from the adolescents were collected.

## Al-Khaizuran Digital Health Intervention

**Figure 2:**
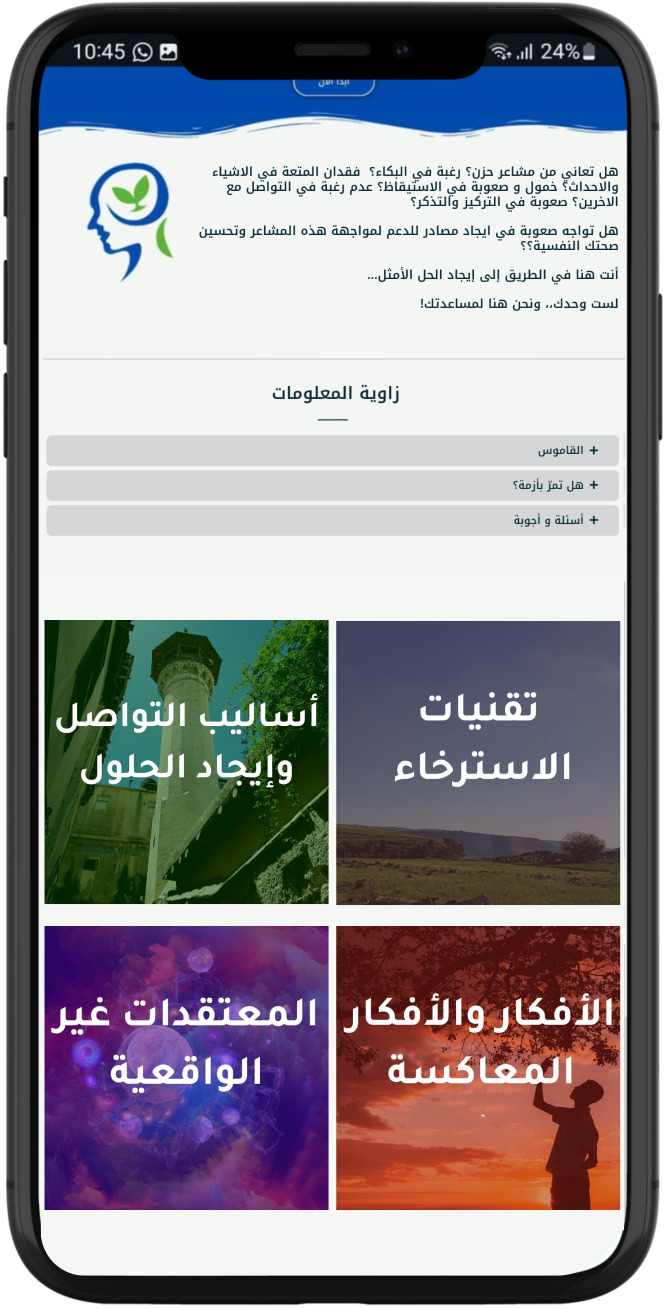
Al-Khaizuran DHI homepage displaying the website's module titles, along with the Frequently Asked Questions section and glossary.

**Figure 3:**
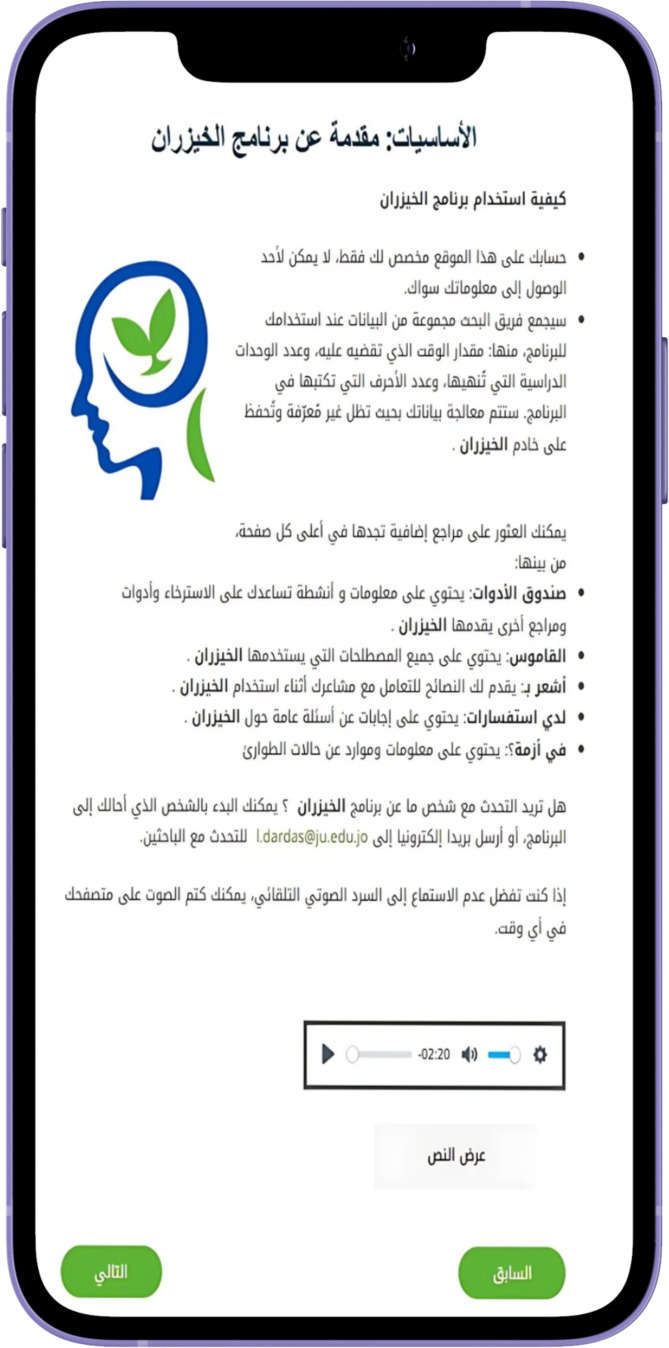
Al-Khaizuran DHI content guide providing detailed instructions on how to navigate the website.

**Figure 4:**
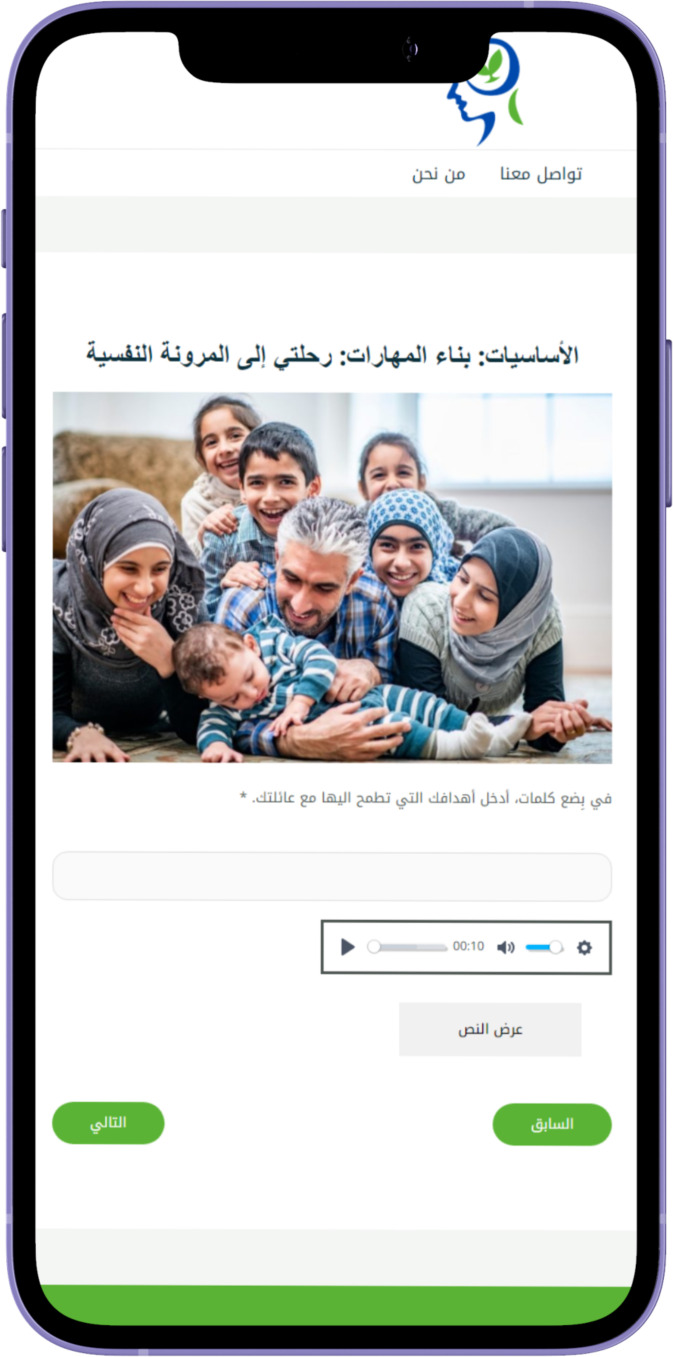
Al-Khaizuran DHI character adaptation.

**Figure 5:**
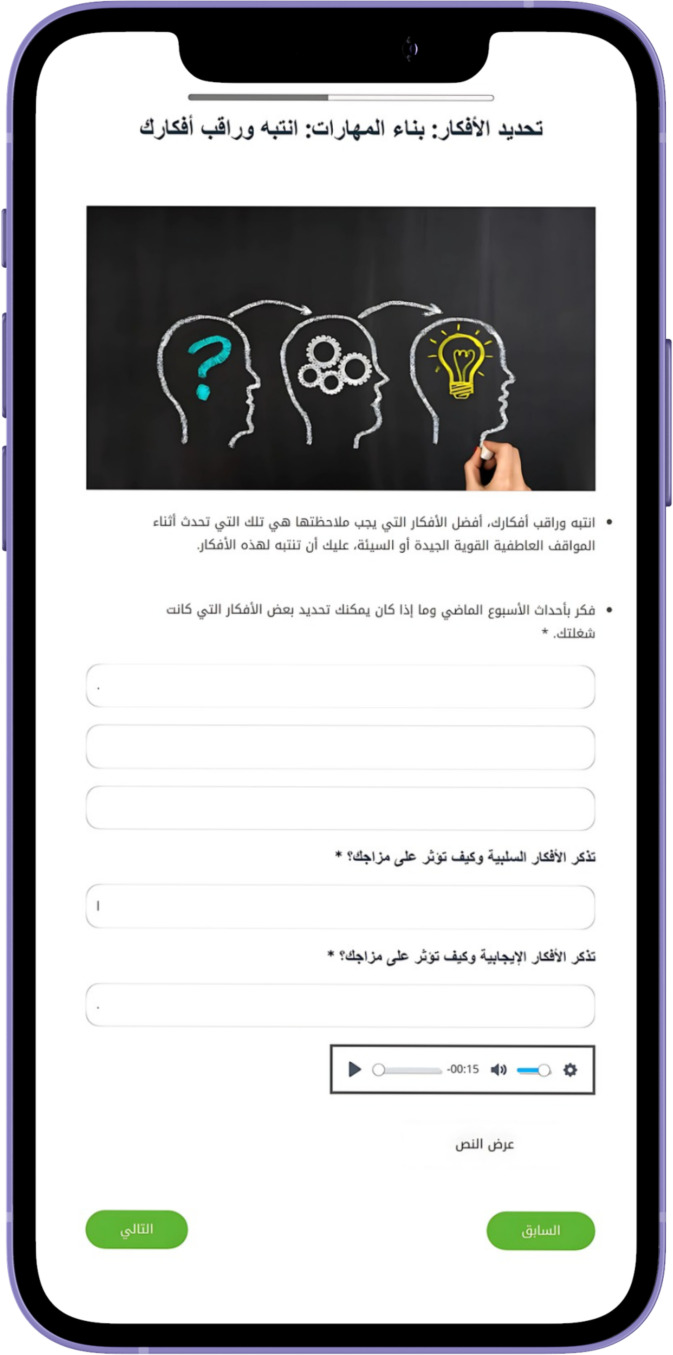
Sample of Al-Khaizuran DHI exercises supported with audiovisuals. The content focuses on how to identify automatic negative thoughts.

**Figure 6:**
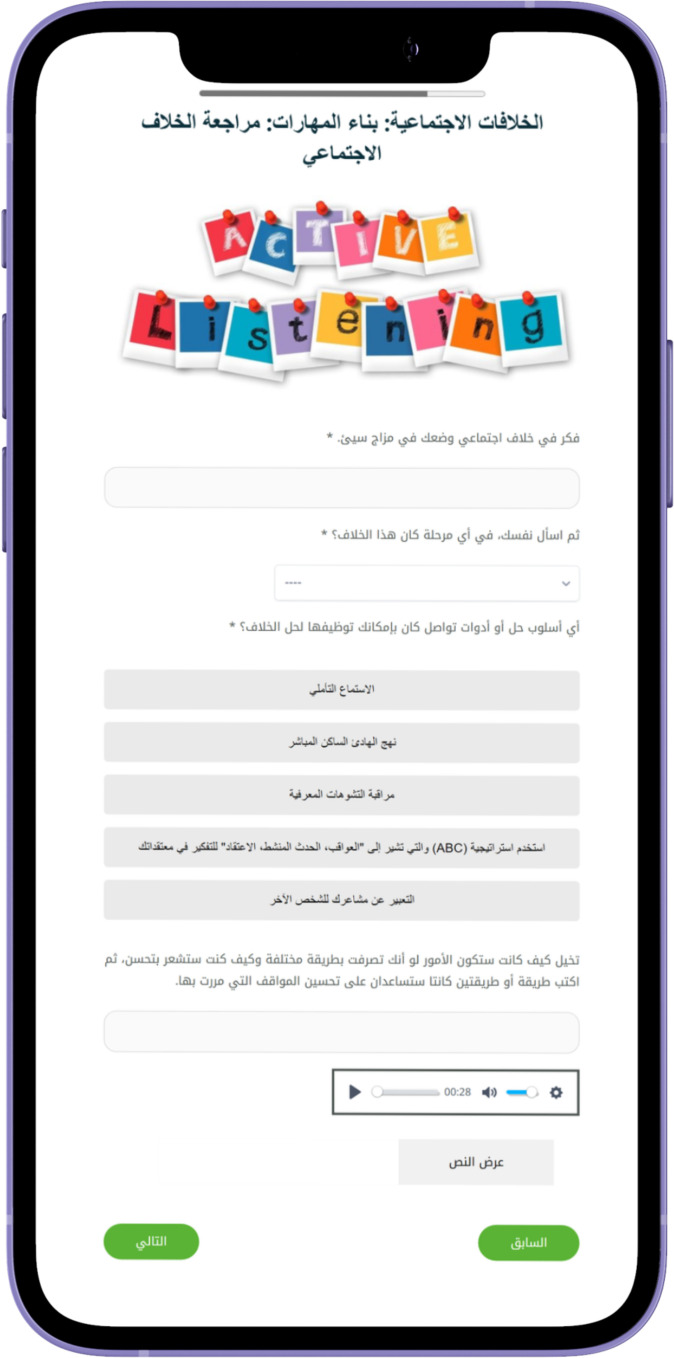
Sample of Al-Khaizuran DHI interactive exercises focused on solving social conflicts.

Al-Khaizuran DHI is a self-paced, 10-module digital program designed to help Jordanian adolescents manage depression symptoms, prevent depressive disorders, and build resilience. The modules are concise and user-friendly, allowing participants to progress at their own speed, accommodating individual schedules and learning preferences. Completion time varies based on personal engagement and pacing. The modules generally follow a consistent structure to facilitate learning and engagement. They begin with an introduction to key concepts, followed by a recap of the previous module and a warm-up exercise to reinforce learning. See **Supplementary Table S1** for the modules content. The main content also includes questions, examples, and discussions, complemented by real-life stories that demonstrate the skills being taught. The modules also include real-life stories that demonstrate the application of the skills taught, making the content relatable. Users then complete exercises, known as Skill Builders, which allow them to apply what they have learned to their own lives. Feedback sections invite users to reflect on the module, while the Wrap-Up recaps the main points and sets the stage for the next module. Additionally, users are encouraged to set new goals and engage in self-training exercises, with a reward system featuring fun activities and a bulletin board for personal interaction, further fostering a sense of community. See **Figures 2**–**6** of the DHI pages.

## The control: school-based group CBT

This school-based group therapy program consisted of eight structured sessions aimed at providing CBT interventions. We used the Coping with Depression: Adolescent Course, which was the foundation for the CBT sections of the CATCH-IT materials (43). The aim was to compare a DHI with a group-based model, both of which are based on the same source manual. The program was designed to address symptoms of depression, anxiety, and general distress through a combination of psychoeducation, mood assessments, and behavioral exercises. Each session builds upon previous ones, progressively equipping participants with skills such as recognizing negative thought patterns, cognitive restructuring, behavioral activation, and problem-solving techniques. The program emphasizes both self-monitoring and practical application of CBT strategies, with a focus on fostering long-term emotional regulation and preventing relapse. Participants were given homework assignments after each session to reinforce the techniques learned, enhancing both individual engagement and therapeutic outcomes. The group sessions were facilitated by a certified educational psychologist who had received specialized training in delivering the training course materials. These sessions were held in designated activity rooms within the same schools, offering a familiar and supportive environment for participants. To ensure the quality and consistency of the intervention, fidelity checks were performed by the leading author to monitor the psychologist’s adherence to the program’s protocols. See **Supplementary Table S2** for the sessions content.

## Randomization

The random allocation sequence was generated using Qualtrics software and integrated into the baseline questionnaire. An independent research coordinator, not involved in the intervention delivery or outcome assessment, enrolled the participants by verifying their eligibility and ensuring completion of the baseline assessment. Once the assessment was completed, Qualtrics automatically assigned participants to either the Al-Khaizuran group or the group CBT group in a 1:1 ratio. See **Supplementary Figure S1**.

## Measures

The Beck Depression Inventory-II (BDI-II) (70) was used to evaluate the presence and intensity of depressive symptoms in participants. The validated Arabic version (63) comprises 21 items, each offering four self-assessment statements covering a two-week period, with scores ranging from 1 to 4. The total depression score, which ranges from 21 to 84, is calculated by summing the responses across all items, with higher scores indicating more severe depression. Based on the total scores, each adolescent’s level of depression is categorized into four distinct ranges: (1) minimal (21 to 34), (2) mild (35 to 40), (3) moderate (41 to 49), and (4) severe (50 to 84). In this study, the Cronbach’s alpha for the scale was.92.

The Connor-Davidson Resilience Scale-Short Form (CD-RISC-10) (71) was used to assess resilience, including personal competence, tenacity, tolerance to negative affect, strengthening effects of stress, positive acceptance of change, secure relationships, control, and spiritual influences. The scale comprises 10 items selected from the original 25-item CD-RISC-25. Respondents’ scores can range from 0 to 40, with responses rated as: 0 = Not true at all, 1 = Rarely true, 2 = Sometimes true, 3 = Often true, and 4 = True nearly all the time. The scale demonstrated good reliability, with α = .81 and ω = .82 (72). In this study, the Cronbach’s alpha was.88.

The Dysfunctional Attitude Scale-Shortened Version (DAS-SV) (73) was used to measure maladaptive cognitive patterns associated with psychopathology, such as those outlined in Beck’s cognitive theory. This scale includes items designed to capture underlying assumptions and beliefs that, when combined with stressors, could lead to clinical symptoms (74, 75). Derived from a factor analysis of the original 100-item DAS (76), it uses a 7-point Likert scale ranging from 1 (totally disagree) to 7 (totally agree). The items reflect rigid and absolute thinking patterns using terms like “always,” “never,” and “must.”

The Preferences for Intervention Scale (77) was used to measure individual preferences for different psychological intervention modalities. The scale includes 16 items that assess participants’ comfort and willingness to engage in various treatment options, such as natural recovery, medication, one-on-one counseling, or group counseling. Participants rated statements, including “I will wait and get over it naturally” and “Use anti-depressant drugs,” on a Likert scale from 1 (definitely acceptable) to 4 (definitely not acceptable). Additional options included consulting a primary care physician or completing a questionnaire online. All items followed the same rating scale as the original (47). This scale is particularly relevant for understanding patient engagement and acceptance of the intervention.

The Behavioral Intention Scale (78) was used to assess participants’ intentions to change their approach to solving everyday problems to mitigate depression risk. The scale includes statements reflecting various stages of consideration and decision-making, such as “I have not given any thought to changing…” and “I have decided to change … and I have a plan.” Participants select the statement that best describes their current position, with higher scores indicating stronger commitment to behavioral change.

The Attitudes Toward Depression Prevention Scale (ATDPS) (44) was utilized to assess participants’ beliefs and dysfunctional attitudes toward depression prevention strategies, such as lifestyle modifications, early intervention, and educational initiatives. This scale consists of multiple items designed to evaluate how participants perceive the significance and effectiveness of these strategies, as well as their readiness to engage in preventive actions critical factors.

The Usefulness, Satisfaction, and Ease of Use Questionnaire (USE) (78) was used to assess the perceived usefulness, ease of use, ease of learning, and satisfaction with the intervention. The scale includes items such as “It gives me more control over the activities in my life,” “Both occasional and regular users would like it,” and “I easily remember how to use it.” Responses were rated on a 1–7 Likert scale ranging from strongly disagree to strongly agree.

The Website Engagement Tool (78) was used to measure the level of engagement in the Al-Khaizuran DHI. This included two scores: total amount of time spent on the site, and total number of modules completed by participants. These components are consistent with prior CATCH-IT research on engagement, which included the same variables (43).

## Tools translation

With the exception of the BDI-II and DASS tools, which have valid and reliable Arabic versions, all other instruments used in this study were translated into Arabic following the WHO guidelines for translation (79). This process involved three key steps: initial translation, cultural adaptation, and validation. The translation emphasized semantic, technical, and conceptual equivalence to ensure that meanings and cultural relevance were preserved. Key informant interviews and focus groups were conducted to refine the tools further, incorporating feedback from diverse participants to address issues of readability and cultural appropriateness. Back translation was then used to verify the accuracy of the translated versions, and revisions were made accordingly.

## Statistical analysis

SPSS version 23.0 was used to conduct the analysis. Categorical variables were presented as frequencies while continuous variables were presented as means ± standard deviations. Differences in patient-centered outcomes pre and post intervention were tested using repeated Measures ANOVA adjusted for gender and age. Differences in post-interventional patient-centered outcomes per intervention were tested using ANCOVA adjusted for gender and age. Multiple multivariate linear regression models were utilized to detect predictors of behavioral intention to prevent depression. A p-value of less than 0.05 was set for statistical significance. For the univariate analysis, normality was checked and all scores were normally distributed. However, due to the small sample size, non-parametric testing was also used, and it showed the same results as their parametric counterparts.

As per the qualitative data, we utilized the approach outlined by Iloabachie et al. (80) for the analysis. This process involved four members of the research team compiling and evaluating the data to identify recurring themes. Grounded theory methodology was applied, with each team member independently reviewing the material. The team then discussed potential themes, and after thorough deliberation, consensus was reached on the key themes, with the belief that thematic saturation had been achieved. Following this, two team members categorized all comments according to the agreed themes. Any differences in categorization were resolved between the two members until they achieved 85% agreement on the final taxonomy. To ensure objectivity and reduce bias, we implemented several strategies. First, the research team was composed of individuals who had no prior involvement with the CATCH-IT program and who worked independently from the intervention’s developer. Second, both raters maintained diaries throughout the study to monitor and mitigate any potential bias. Finally, to further assess bias in developing the taxonomy, we reviewed the classification system and the comments with one interviewer to confirm that the taxonomy and comment classification aligned with their interview experiences.

## Results

We included a total of 109 participants with a mean age of 16.1 ± 0.7 years and male-to-female ratio of 0.78-to-1. The majority of participants did not report chronic illnesses (92.8%) nor sought psychiatric help (88.7%). High school was the highest degree of education for most fathers (50.6%) and mothers (48.1%) of included children. Participants’ characteristics are included in **Table 2**.

**Table 2:** Sociodemographic characteristics of the included sample (n = 117).

		Al-Khaizuran DHI		Group CBT		
Variable	Category	N	%	N	%	p
Gender	Male	26	48.1%	25	46.3%	0.847
	Female	28	51.9%	29	53.7%	
Age	15 years	0	0.0%	25	46.3%	<0.001
	16 years	54	98.2%	2	3.7%	
	17 years	1	1.8%	27	50.0%	
Chronic Illness Status	No	44	91.7%	45	93.8%	0.695
	Yes	4	8.3%	3	6.3%	
Sought psychological help	No	45	88.2%	40	88.9%	0.920
	Yes	6	11.8%	5	11.1%	
Father’s educational level	Primary	4	9.8%	11	31.4%	0.046
	High school	21	51.2%	18	51.4%	
	Diploma	8	19.5%	2	5.7%	
	Bachelor’s Degree	8	19.5%	4	11.4%	
Father’s educational level	Primary	3	7.3%	4	11.4%	0.628
	High school	18	43.9%	19	54.3%	
	Diploma	9	22.0%	5	14.3%	
	Bachelor’s Degree	11	26.8%	7	20.0%	
Monthly income	Less than 300	10	29.4%	10	24.4%	0.302
	300 to 500	12	35.3%	19	46.3%	
	501 to 1000	7	20.6%	11	26.8%	
	1001 to 1500	2	5.9%	0	0.0%	
	More than 1500	3	8.8%	1	2.4%	

### Aim 1: evaluate the feasibility and cultural acceptability of Al-Khaizuran DHI among Jordanian adolescents, and compare its acceptability to targeted school-based group CBT

Both qualitative and quantitative data were collected to evaluate the feasibility and cultural acceptability of Al-Khaizuran DHI among Jordanian adolescents. This approach also allowed for a comparison of its acceptability to targeted school-based group CBT. Below are results of the analyses of the semi-structured interviews with participants arranged around two main themes: content and language, and accessibility and engagement.

#### The content and language

The content and language used on the website received mixed feedback from the students, highlighting areas of both strength and improvement. Many appreciated the interactive elements, such as being asked to recall personal experiences, and found the exercises and activities, like relaxation techniques, to be highly beneficial. The simplicity and positivity of the website’s ideas were well-received, with students expressing that the content was generally easy to understand and engage with. However, several students noted that the language used was relatively formal, suggesting that a mix of colloquial and formal Arabic would make the content more accessible and relatable.

“*I would have preferred the language to be colloquial rather than standard Arabic, as at some points I was listening to boring news!*”

There was also a recurring concern about the amount of text, with several students reporting a desire for a more personalized approach, where users could access only the sections most relevant to them to reduce the need to sift through extensive text. The audio features of the website were praised for making the content more engaging.

“*Hearing the text helped me a lot, without the audio clips that read the text the site would be boring.*”

Several students appreciated the realism of the stories, noting that they reflected real-life issues and provided useful insights. These students found the narratives impactful, with some even drawing parallels between the stories and their own experiences. However, a few students expressed a desire for more detailed narratives, preferring to have the full context and background to fully grasp the lessons being conveyed.

“*The stories were brief, but I prefer to know all the details, even the smallest ones, as they might capture my interest more.*”

“*I liked the stories, and some had a lasting impact that I always remember. I could even relate some of the stories to myself.*”

#### Accessibility and engagement

The interviews revealed a wide range of experiences regarding accessibility and engagement with the website. Some students were able to incorporate the site into their daily routines, spending between 15 minutes to an hour per session and finding it beneficial enough to consider making it a regular part of their lives. These students reported using the site consistently for about a week or more, with a few indicating that their engagement would be higher if they had their own devices. One student mentioned using the site for five to seven days, dedicating an hour each day. However, they noted that they are unlikely to continue this routine due to not having access to a personal device. Another student said that they initially used the site daily for two weeks but did not continue due to a lack of motivation, despite benefiting from the sections they completed. Almost all students reported that they wish they could discuss the website content consistently with a mental health professional.

Device accessibility emerged as a significant barrier for several students. Those who did not have a personal device, and instead used a parent’s or sibling’s device, reported discomfort and concerns about privacy, which hindered their engagement with the site. For instance, one student noted that they could only use their mother’s phone, which limited their ability to participate in the site’s activities consistently. Similarly, another student who shared a device with their father mentioned that using the site was not comfortable due to the lack of privacy, which discouraged them from continuing despite recognizing its benefits. These students suggested that their engagement would be higher if they had their own devices.

“*I didn’t use the exercises on the site, because the mobile that I enter from on the site is for my mother, so I didn’t have much time*“

“*My problem was that I didn’t have my own device to use the site, I was using my parents’ device but this was never comfortable, I didn’t want anyone to read what I was writing on the site.*”

Time constraints also played a role in how students engaged with the website. Some students with more free time were able to spend extended periods on the site, sometimes using it for over an hour per session. In contrast, those with after-school responsibilities, such as work or family obligations, found it challenging to find time to engage with the site regularly. One student, who worked after school, noted that they were unable to find sufficient time to use the site, which limited their ability to fully benefit from its content. All the students who were interviewed suggested that time be allocated at school to use the site.

“*I had a lot of free time that part of it filled the use of the site, I was happy to fill my free time with something useful*”

“*I think it is necessary to allocate appropriate time daily or weekly at school to use the site*”

The desire for a more modern and interactive design was echoed by several students who found the current layout too rigid and static. They expressed a preference for a more dynamic and engaging interface, comparable to popular social media platforms, which they felt would make the site more appealing and user-friendly.

“*If I were to change something, it would be the way the text is presented. I feel like there should be more colors and images.*”

“*The colors used were easy on the eyes. I love the color green; it makes me feel optimistic.*”

Overall, while the website’s usability was generally well-received, there was a clear demand for enhancements in its design and interactivity to better meet the expectations of its young users.

#### Quantitative data

Based on the questionnaire assessing usefulness, satisfaction, and ease of use, participants shared several notable observations about Al-Khaizuran DHI. Around half of the participants felt the program helped them be effective (52.9%) and empowered them (54.0%). Similar proportions found it simple to use (54%), flexible (51.0%), consistent (46.0%), quick to learn (57.1%), intuitive (55.1%), and pleasant to use (52.0%). Additionally, 51.0% reported being satisfied with the program, and 51.0% indicated they would recommend it to others. However, a considerable number of responses were neutral, categorized as “Neither” (see **Table 3**). These neutral responses may not necessarily signify dissatisfaction. Further analysis of usage data revealed that many participants who selected neutral responses had completed less than 60% of the program.

**Table 3:** Responses of participants to the usefulness, satisfaction, and ease of use questionnaire.

Statement	Strongly Disagree		Disagree		Neither		Agree		Strongly Agree	
	N	%	N	%	N	%	N	%	N	%
It helps me be more effective.	7	13.7%	2	3.9%	15	29.4%	20	39.2%	7	13.7%
It helps me be more productive.	4	8.0%	3	6.0%	21	42.0%	21	42.0%	1	2.0%
It is useful.	2	4.2%	3	6.3%	17	35.4%	15	31.3%	11	22.9%
It gives me more control over the activities in my life.	2	4.0%	3	6.0%	18	36.0%	20	40.0%	7	14.0%
It makes the things I want to accomplish easier to get done.	4	7.8%	4	7.8%	20	39.2%	15	29.4%	8	15.7%
It saves me time when I use it.	3	6.0%	4	8.0%	24	48.0%	10	20.0%	9	18.0%
It meets my needs.	4	8.2%	8	16.3%	21	42.9%	9	18.4%	7	14.3%
It does everything I would expect it to do.	3	6.0%	5	10.0%	20	40.0%	9	18.0%	13	26.0%
It is easy to use.	3	5.9%	3	5.9%	16	31.4%	15	29.4%	14	27.5%
It is simple to use.	3	6.0%	5	10.0%	15	30.0%	16	32.0%	11	22.0%
It requires the fewest steps possible to accomplish what I want to do with it.	4	8.3%	8	16.7%	12	25.0%	13	27.1%	11	22.9%
It is flexible	3	5.9%	5	9.8%	17	33.3%	21	41.2%	5	9.8%
Using it is effortless.	1	2.0%	8	16.3%	17	34.7%	16	32.7%	7	14.3%
I can use it without written instructions.	4	8.0%	6	12.0%	20	40.0%	12	24.0%	8	16.0%
I don't notice any inconsistencies as I use it.	3	6.0%	6	12.0%	18	36.0%	19	38.0%	4	8.0%
Both occasional and regular users would like it.	2	4.0%	12	24.0%	22	44.0%	9	18.0%	5	10.0%
I can recover from mistakes quickly and easily.	1	2.0%	7	14.0%	22	44.0%	13	26.0%	7	14.0%
I can use it successfully every time.	3	5.9%	7	13.7%	14	27.5%	16	31.4%	11	21.6%
I learned to use it quickly.	6	12.2%	3	6.1%	12	24.5%	17	34.7%	11	22.4%
I easily remember how to use it.	4	8.2%	5	10.2%	13	26.5%	17	34.7%	10	20.4%
I quickly became skillful with it.	3	6.3%	7	14.6%	16	33.3%	10	20.8%	12	25.0%
I am satisfied with it.	5	10.2%	4	8.2%	15	30.6%	19	38.8%	6	12.2%
I would recommend it to a friend.	4	8.2%	8	16.3%	12	24.5%	18	36.7%	7	14.3%
It is fun to use.	6	12.0%	5	10.0%	13	26.0%	16	32.0%	10	20.0%
It works the way I want it to work.	2	4.1%	10	20.4%	16	32.7%	14	28.6%	7	14.3%
It is wonderful.	5	10.0%	6	12.0%	13	26.0%	18	36.0%	8	16.0%
I feel I need to have it.	5	10.0%	11	22.0%	14	28.0%	16	32.0%	4	8.0%
It is pleasant to use.	4	8.0%	7	14.0%	13	26.0%	19	38.0%	7	14.0%

#### Comparing the acceptability of Al-Khaizuran DHI to the school-based group CBT

With regards to data completion, the results showed notable differences in engagement patterns. For Al-Khaizuran DHI, a significant portion of participants completed 6 to 8 modules out of 10, with the highest percentage (27.3%) completing 6 modules, followed by 18.2% completing 7 modules, and 14.5% completing 9 modules. In contrast, Group CBT participants showed a concentrated completion trend in the mid-range sessions, with 22.2%, 25.9%, and 24.1% completing 4, 5, and 6 sessions, respectively. Both interventions had no participants completing 0 or 1 session (see **Table 4**).

**Table 4:** Participants adherence data.

Al-Khaizuran DHI		
Number of Sessions Completed	Participants (n)	Participants (%)
0	0	0.0
1	0	0.0
2	0	0.0
3	3	5.5
4	4	7.3
5	6	10.9
6	15	27.3
7	10	18.2
8	5	9.1
9	8	14.5
10	4	7.3

Group CBT		
Number of Sessions Completed	Participants (n)	Participants (%)
0	0	0.0
1	0	0.0
2	2	3.7
3	4	7.4
4	12	22.2
5	14	25.9
6	13	24.1
7	6	11.1
8	3	5.6

Despite initial challenges in adopting the Al-Khaizuran DHI, it was ultimately received more positively than the school-based group CBT. Post-intervention data (see **Supplementary Table S3**) indicate that 40% of participants found the option of completing an online program with their primary care provider (PCP) as “definitely acceptable,” a significant increase from 14.4% in the pre-intervention phase (**Supplementary Table S4**). Additionally, preferences for one-on-one counseling increased, with 24.8% rating it as “definitely acceptable” post-intervention compared to 12.4% pre-intervention. In contrast, support for group-based interventions, particularly group counseling, saw a notable decline. Pre-intervention, 12.4% of participants found group meetings with peers “definitely acceptable,” while this dropped to 11.8% post-intervention. Moreover, preferences for group counseling with six or more patients decreased significantly, with 44.1% finding it “definitely not acceptable” after the intervention, compared to 25.8% before.

Prior to the intervention, participants showed limited support for several depression prevention methods, such as counseling (35.8%), consulting healthcare providers for mental illness solutions (39.3%), engaging in exercise (49.5%), attending coping workshops (51.1%), or taking prescribed medications (39.2%) (see **Supplementary Table S4**). However, after completing the interventions, participants from both groups exhibited more favorable attitudes toward a variety of intervention strategies. Notably, there was a stronger preference for one-on-one counseling, while interest in group-based interventions appeared to diminish (see **Supplementary Table S3**).

### Aim 2: evaluate the comparative effectiveness of Al-Khaizuran and school-based group CBT in preventing the onset of depressive episodes and improving other patient-centered outcomes (depressive symptoms, resiliency, and intervention attitudes and preferences) among Jordanian adolescents

A total of 55 participants were randomly assigned to Al-Khaizuran DHI, while 54 were assigned to the group CBT. Following the completion of the pre and post testing, the depression scores among participants in both groups demonstrated no significant difference (all p>0.05) compared to baseline scores. However, participants who underwent Al-Khaizuran DHI showed a trend toward a reduction in dysfunctional attitudes toward depression (p=0.054). On the contrary, participants in the group CBT demonstrated lower resilience scores (p = 0.031) and worse perceptions of intervention (p = 0.019) (Refer to **Table 5**). Post-interventional scores between the two groups remained statistically indifferent for the exception of resilience scores, which were significantly lower for the CBT group (p = 0.026) (Refer to **Table 6**).

**Table 5:** Differences in patient-centered outcomes pre and post intervention.

	Al-Khaizuran DHI		
	Pre-intervention	Post-intervention	p-value
Beck’s Depression Inventory	40.3 ± 10.6	36.8 ± 16.4	0.333
Connor-Davidson Resilience Scale	26.9 ± 61.2	25.3 ± 9.9	0.269
Dysfunctional Attitude Scale	17.7 ± 6.3	20.1 ± 8.7	0.054
Preferences for Intervention Scale	37.9 ± 9.5	42.4 ± 12.0	0.854
	CBT		
Beck’s Depression Inventory	39.6 ± 11.1	38.8 ± 13.8	0.244
Connor-Davidson Resilience Scale	24.6 ± 7.4	20.8 ± 8.7	0.031
Dysfunctional Attitude Scale	18.6 ± 4.7	18.0 ± 5.9	0.342
Preferences for Intervention Scale	36.8 ± 8.7	43.4 ± 11.6	0.019

*Repeated Measures ANOVA adjusted for gender and age.

**Table 6:** Differences in post-interventional patient-centered outcomes per intervention.

	Al-Khaizuran DHI	Group CBT	p-value
Beck’s Depression Inventory	36.0 ± 15.7	39.3 ± 13.6	0.277
Connor-Davidson Resilience Scale	25.3 ± 9.7	20.7 ± 9.0	0.026
Dysfunctional Attitude Scale	20.1 ± 8.5	18.8 ± 6.5	0.319
Preferences for Intervention Scale	42.4 ± 11.8	43.3 ± 11.4	0.708

*ANCOVA adjusted for gender and age.

### Secondary aim 1: to explore the role of adolescents’ attitudes and beliefs regarding the intervention’s effectiveness as predictors of their intention to adopt preventive behaviors and engage in behavioral changes

We explored predictors of behavioral intention both pre- and post-intervention using multivariate linear regression. The analysis focuses on various beliefs, attitudes, and perceptions related to the depression prevention intervention and how these factors influenced participants’ intention to engage in preventive behaviors before and after completing the intervention (see **Table 7**). Before the intervention, several beliefs about the effectiveness of a depression prevention program, such as “A depression prevention intervention makes sense to me” and “I believe that I can protect myself from depression through a prevention intervention,” were not statistically significant predictors of behavioral intention (p = 0.151 and p = 0.220, respectively). These results suggest that, initially, participants were either unsure about or had neutral attitudes toward the importance and relevance of the intervention in preventing depression. Additionally, attitudes toward the ease of going through the intervention, including “I think going through this prevention intervention will be easy,” did not significantly influence behavioral intention pre-intervention (p = 0.594). However, following the intervention, the results showed notable changes in the predictors of behavioral intention. For example, beliefs such as “A depression prevention intervention could prevent further depression” and “I believe a prevention intervention will help me to stay healthy” became significant predictors of behavioral intention (p = 0.022 and p = 0.03, respectively). This shift indicates that participants’ confidence in the effectiveness of the intervention improved after experiencing the program, which in turn positively impacted their intention to engage in preventive behaviors.

**Table 7:** Predictors of behavioral intention.

Attitudes Toward Depression Prevention	Pre-intervention				Post-intervention			
	B	L95%CI	U95%CI	p-value	B	L95%CI	U95%CI	p-value
Beliefs about intervention								
A depression prevention intervention makes sense to me.	0.230	-0.097	0.613	0.151	0.073	-0.691	0.881	0.808
I believe that I can protect myself from depression through a depression prevention intervention.	0.203	-0.152	0.644	0.220	0.119	-0.597	0.905	0.681
I think that participating in intervention like this is important.	-0.062	-0.472	0.326	0.716	-0.139	-0.941	0.579	0.633
I believe that when depression is found early it can be prevented.	-0.168	-0.534	0.159	0.284	0.303	-0.500	1.255	0.390
I believe that a depression prevention intervention could prevent further depression.	-0.068	-0.439	0.271	0.637	0.45	0.2	0.7	0.022
I believe a depression prevention intervention Will help me to be healthy.	-0.328	-0.759	0.037	0.075	0.4	0.1	0.7	0.03
I think would be better of without a depression prevention intervention.	-0.127	-0.424	0.170	0.396	-0.106	-0.791	0.519	0.677
Attitudes toward behavior								
I think going through a depression prevention intervention is too much trouble.	-0.097	-0.362	0.172	0.479	-0.45	-0.7	-0.2	0.015
I think the benefits of a depression prevention intervention overweigh any difficulty.	0.159	-0.546	1.017	0.546	0.324	0.058	0.590	0.018
Going through a depression prevention intervention is too embarrassing.	-0.5	-0.8	-0.2	0.022	-0.003	-0.554	0.545	0.987
Perceived social norms								
Members of my immediate family support my going through a depression prevention program.	0.173	-0.098	0.414	0.223	0.058	-0.519	0.665	0.805
I want to please members of my immediate family.	0.051	-0.261	0.361	0.749	0.635	-0.100	1.568	0.083
I want to do what the physicians want me to do About a depression prevention intervention.	0.069	-0.303	0.463	0.677	-0.317	-1.298	0.477	0.356
I think that the physicians want me to do a depression prevention intervention.	-0.130	-0.456	0.196	0.428	-0.177	-1.280	0.823	0.663
My close friends support my going through a depression prevention program.	-0.146	-0.386	0.110	0.269	-0.250	-1.045	0.389	0.362
I want to please my close friends.	-0.215	-0.513	0.077	0.145	-0.126	-0.742	0.433	0.598
I trust my doctor.	0.007	-0.268	0.281	0.962	0.5	0.2	0.8	0.025
Self-efficacy								
I think going through this depression prevention will be easy.	-0.089	-0.450	0.260	0.594	-0.45	0.1	0.8	0.035
Arranging my schedule for depression prevention intervention will be easy.	0.130	-0.23	0.498	0.464	-0.4	0.1	0.7	0.04
Demographics								
Participant Age	0.309	-0.008	1.126	0.053	0.183	-0.68	1.503	0.451
Gender	-0.024	-0.796	0.685	0.881	0.102	-0.951	1.569	0.623
Group (CBT or Website)	NA	NA	NA	NA	-0.074	-1.262	0.811	0.663

Additionally, while perceived difficulty in going through the intervention (“I think going through this prevention intervention will be easy”) was not a significant predictor pre-intervention, it became significant post-intervention, but with a negative coefficient (B = -0.45, p = 0.035). This suggests that participants found the intervention more challenging than anticipated, which may have dampened their intentions to fully adhere to the intervention.

Another significant post-intervention predictor was the perceived benefit of the intervention relative to any difficulties encountered. Participants who believed that “The benefits of a prevention intervention outweigh any difficulties” showed a positive association with behavioral intention (B = 0.324, p = 0.018). This highlights that those who felt the intervention was worth the effort were more likely to follow through with their intentions to engage in preventive behaviors. In summary, post-intervention, participants showed a stronger belief in the intervention’s ability to prevent depression and maintain their health, which positively influenced their behavioral intentions. However, increased perceptions of the difficulty of the intervention also emerged as a challenge that could affect adherence.

## Discussion

This study evaluated the feasibility, cultural acceptability, and effectiveness of Al-Khaizuran DHI among Jordanian adolescents, with comparisons to school-based group CBT. The findings provide valuable insights into the intervention’s potential to address depressive symptoms and build resilience in this population. Additionally, the study explored adolescents’ attitudes and beliefs about the intervention as predictors of their intention to adopt preventive behaviors and engage in behavioral changes. The discussion focuses on interpreting these findings in the context of the study’s aims, addressing both the strengths and limitations of the interventions, and considering implications for future research and practice.

The findings were promising, especially in relation to the feasibility and acceptability of the Al-Khaizuran DHI. Over half of the participants reported that the intervention was effective, empowering, and easy to use, with 51% expressing satisfaction and willingness to recommend it to others. Qualitative data further supported these positive outcomes, providing detailed insights into participants’ experiences with the program, including the value of its flexibility and user-friendliness.

Many appreciated the interactive elements, such as recalling personal experiences and engaging in relaxation exercises, which were viewed as practical and beneficial. The audio features were highlighted as a key strength, enhancing engagement and making the site more user-friendly. Additionally, the realism of the stories resonated with participants, who found them relatable and impactful. Several students expressed a willingness to incorporate the program into their daily routines.

Our implemented version of the CATCH-IT program (i.e., Al-Khaizuran) did not induce significant changes in depression scores among participants. A number of factors may contribute to this result. First, the online nature of the intervention, while generally accessible, may have lacked the personal engagement and direct feedback that participants need. The lesson learned from this trial is that a blended approach, which combines the flexibility of digital tools with periodic in-person support maybe more efficacious. This approach was suggested and supported by participants themselves, as evidenced in their self-report assessment tools and qualitative interviews. Additionally, interviews highlighted the participants’ desire to discuss the content with a specialist, emphasizing the need for real-time interaction and personalized guidance. This hybrid model could optimize engagement and efficacy across diverse participant preferences.

Although Al-Khaizuran DHI was culturally adapted and translated for the target population, it is possible that the duration of the program may have been too short to elicit significant changes in depression levels. Participants lack self-motivation to engage with the program and lack of incentive to engage longer with the program modules are also possible challenges (80). It also remains possible that the content of the program itself did not adequately engaged participants in a way that leads to measurable changes in depression scores. For example, the absence of real-time, individualized feedback within the DHI may have reduced its effectiveness. Further, participant feedback from interviews provided valuable insights into how the DHI could be improved to better meet the needs of its adolescent users. Several students expressed a preference for a more dynamic and modern design, including vibrant visuals and a layout similar to social media platforms, to make the site more user-friendly. The language used in the intervention was another key area for improvement, with participants suggesting a mix of colloquial and formal Arabic to enhance accessibility and relatability. Additionally, concerns about the extensive text content led to a recommendation for a more personalized approach, allowing users to focus on sections most relevant to their needs. Notably, during the adaptation process, the original content was reduced from 14 to 10 modules based on initial feedback and general impressions from potential participants. However, feedback from the current study suggests this reduction was not sufficient to address the concerns about the volume and complexity of the material. Incorporating these improvements will enhance the intervention’s relevance and effectiveness within the sociocultural context of the target population.

Another critical point raised by the participants during the interviews is the reliance on personal smartphones for accessing the intervention. Adolescents have encountered issues such as limited access to devices, or privacy concerns when using shared family phones, which could hinder full engagement with the intervention. Addressing these technological barriers in future iterations will require proactive parental engagement, as providing devices or ensuring private access cannot proceed without parental approval in the Jordanian context. Parental involvement could help facilitate access and support adolescents’ engagement with the intervention, thereby enhancing its overall effectiveness. Moreover, understanding how adolescents interact with module content and their preferences for tailored support, alongside parental insights, may guide refinements to better meet the needs of both the target population and their families.

In assessing the comparative effectiveness, while depression scores between the Al-Khaizuran and CBT groups did not significantly differ post-intervention, participants in the Al-Khaizuran group showed a trend toward reduced dysfunctional attitudes toward depression interventions. On the other hand, those in the group CBT reported lower resilience and a poorer perception of the intervention. While this could emphasize the potential for Al-Khaizuran to be a more acceptable and engaging option for depression prevention among Jordanian adolescents, the observed decline in the scores among group CBT participants may be explained by the influence of group dynamics, such as social conformity and social comparison. Adolescents are particularly susceptible to peer influences, and the pressure to conform to group norms can lead to internal conflicts or reduced self-esteem when personal beliefs and group standards clash (81). Similarly, social comparison, a common phenomenon in group settings, may result in feelings of inadequacy when participants perceive themselves as less competent than their peers, thereby negatively affecting their resilience (82). These effects are particularly pronounced during adolescence, a developmental period marked by heightened sensitivity to peer evaluation and influence (83). Additionally, group settings can introduce stressors such as fear of negative evaluation or the need to conform, which may counteract the intended benefits of resilience-building activities (84). Understanding these mechanisms highlights the importance of carefully structuring group interventions to promote positive peer interactions while minimizing potential barriers to resilience enhancement.

Another issue worth mentioning is that although the Al-Khaizuran DHI and group CBT interventions are grounded in the same foundational manual, their delivery formats—self-guided digital versus in-person group sessions—likely introduced variations in participant engagement and adherence. These differences could have influenced the comparability of outcomes, as participants may have responded differently to the interventions based on their personal preferences for learning and interaction styles. For instance, the DHI offered flexibility and autonomy, allowing participants to engage with the content at their own pace and in their own time. However, this format may have been less effective for individuals requiring more structured guidance or real-time feedback, potentially limiting its impact on depression scores. Conversely, the group CBT sessions provided direct interaction with a facilitator, which may have been beneficial for building resilience through peer support but could have been less appealing to participants who preferred privacy or individualized approaches. We believe that in the next phase of the project, incorporating metrics to directly compare adherence and engagement patterns between different modes can enhance our understanding of how delivery format influences the effectiveness of depression prevention interventions.

As per the other study outcomes, our study revealed that post-intervention, beliefs about the effectiveness of the depression prevention program significantly predicted behavioral intention, with participants showing greater confidence that the intervention could prevent further depression and improve health. However, perceptions of the difficulty in completing the program emerged as a barrier, negatively affecting intention. Those who believed the benefits outweighed the challenges were more likely to follow through with preventive behaviors. These findings parallel those from other studies, such as those by Van Voorhees et al. (57, 58), which demonstrated that beliefs and attitudes toward an intervention, rather than social norms, are the strongest predictors of intention (57, 58). Furthermore, preferences for individualized and blended interventions, as seen in this study, reflect a broader trend favoring more personalized approaches over traditional group-based methods (47), with support for group counseling significantly declining post-intervention. These findings underscore the importance of tailoring interventions to adolescents’ preferences and beliefs to enhance engagement and adherence, reinforcing the potential of digital health interventions in this demographic (43). Iloabachie et al. (80) found that adolescents subjected to the CATCH-IT program demonstrated significantly higher positive attitudes toward the effectiveness of treatment modalities, particularly, online interventions, and toward their self-efficacy in changing the course of depression. In our study, participants initially expressed varied preferences for different treatment modalities. However, as the study progressed, these preferences shifted toward a strong favor for one-on-one counseling and guided e-mental health programs. Participants also developed positive attitudes toward a range of supportive approaches, including individual counseling, physical exercise, religious groups, and coping workshops, all of which were viewed as effective in alleviating the impact of depression. Despite this, the initial interest in group therapy or fully self-paced interventions diminished, with many participants expressing a general aversion to these modalities. This highlights a clear preference for more personalized and structured forms of support. This phenomenon is not deviant from what is observed within the literature. Participants’ attitudes toward interventions of this kind appear to fluctuate throughout the course of intervention (85). Moreover, it is noted that participants’ skepticism may be a net result of their inability to transform their newfound knowledge into practical behavioral changes; thus, professional and parental involvement may be necessary to guide pediatric participants into adopting a more active stance toward their mental health.

## Limitations

While this study provides valuable insights, several limitations should be acknowledged to contextualize the findings and guide future research. The introduction of adolescent-parent pairs may improve adherence to program and enable proper behavioral change. We should knowledge the narrow age range of participants, which was restricted to adolescents aged 15-17. Although the targeted population was school adolescents aged 13-17, the sample was limited to those for whom consent and assent were secured. Notably, 32 eligible adolescents could not participate because their parents did not consent to their involvement. This restriction potentially limits the generalizability of the findings to younger adolescents within the broader target age range. Parental engagement may help to include a more representative sample encompassing the entire target age group to enhance the applicability of the results. Future similar programs should not only account for baseline levels of mental illness or previous treatments, moderating factors effect (86, 87) such as parental mental health, relationships, socio-economic status, perceived body image, hopelessness, and religiosity to name a few must be accounted for during data collection and data analysis planning. Gladstone (88) supports this phenomenon as they demonstrate that participants with parents with high levels of depression experienced greater improvements in anxiety control, a finding that is in contrast with prior research suggesting that parental depressive symptoms are associated with poorer outcomes for affected children (89, 90). Similarly, participants with better connections to their primary care physicians or who are able to externalize their symptoms benefited greatly from CATCH-IT compared to traditional health education (91). The latter is in line with this study as participants with less ability to externalize their concerns are less likely to match the high demand of self-management the CATCH-IT program entails. Finally, researchers should strive to measures fluctuations in participants’ frustration or boredom as means to improve intervention and tailor its characteristics per the needs of active individuals.

## Conclusion

The findings from this study underscore critical insights into the feasibility, cultural relevance, and effectiveness of the Al-Khaizuran DHI for Jordanian adolescents. The study revealed that while culturally adapted digital interventions can significantly enhance the accessibility and acceptability of mental health care, their success depends heavily on overcoming structural and contextual barriers. First, the results demonstrated that Al-Khaizuran DHI is a feasible and culturally acceptable intervention, with participants highlighting its relevance and engagement potential. However, challenges such as limited access to personal devices, privacy concerns, and participants’ reliance on shared family resources emerged as significant barriers to consistent engagement. These barriers emphasize the need for proactive parental involvement and institutional support, such as providing private access points in schools, to ensure equitable participation. Second, the comparative outcomes between Al-Khaizuran DHI and school-based group CBT highlighted distinct advantages of digital interventions, particularly in fostering autonomy and reducing dysfunctional attitudes toward depression. Nevertheless, it remains equally important to incorporate mechanisms to sustain user motivation and resilience-building through tailored support. Third, the study findings emphasize that cultural adaptation, while critical, must extend beyond content translation to include design and delivery mechanisms that resonate with adolescents’ lived experiences. Participants expressed a preference for more personalized and interactive features, such as dynamic layouts and colloquial language, which could significantly improve engagement. Furthermore, the intervention’s focus on user-driven engagement revealed that adolescents often lack self-motivation to fully benefit from self-guided modules, underscoring the importance of embedding incentives and real-time feedback mechanisms. We are now entering Phase 7 of the project, where we will adapt the program based on these findings and work toward implementing it on a larger scale. Additionally, efforts will be made to integrate parental involvement and professional support, which were identified as potential moderators of treatment effectiveness. In conclusion, the Al-Khaizuran DHI exemplifies the potential of culturally adapted digital interventions in bridging mental health care gaps in resource-limited settings. However, its effectiveness is contingent upon addressing barriers to access, enhancing program interactivity, and integrating hybrid support systems that combine digital tools with in-person guidance. These conclusions provide a roadmap for refining Al-Khaizuran and scaling similar interventions to meet the mental health needs of adolescents in Jordan and beyond.

## Data Availability

The datasets presented in this study can be found in online repositories. The names of the repository/repositories and accession number(s) can be found below: https://doi.org/10.6084/m9.figshare.27328200.v1.

## Supplementary material

Supplementary Figure 1 CONSORT flow diagram.

Supplementary Table S1 Al-Khaizuran Modules content.

Supplementary Table S2 Group CBT sessions content.

Supplementary Table S3 Post-intervention responses of participants to the preferences for intervention scale.

Supplementary Table S4 Pre-intervention responses of participants to the preferences for intervention scale.
