# Supplementary material for: Validating a digital depression prevention program for adolescents in Jordan: cultural adaptation and user testing in a randomized controlled trial
Source: Front Psychiatry. 2025 Feb 12;16:1529006. doi: 10.3389/fpsyt.2025.1529006 (PMC11860973; doi:10.3389/fpsyt.2025.1529006)
Supplement: Supplementary Figure 1 — CONSORT flow diagram. [file SupplementaryFile1.zip › Supplementary tables 1-4.docx]

**Table S1**: Al-Khaizuran Modules content

| **Module** | **Title** | **Content Summary** |
| --- | --- | --- |
| **1** | **The Basics** | Introduces resilience, goal-setting, and coping strategies to help users navigate challenges in life. It encourages reflection on future goals while highlighting how depression can hinder |
| **2** | **Understanding Behavior** | Focuses on the "outside-in" approach, teaching users how their environment and behaviors impact their mood. The goal is to help them identify patterns between actions and emotions to manage their mental health better. |
| **3** | **Changing Behaviors** | Users learn to change their actions to improve their mood by altering their environment and behavior, fostering a greater sense of control and accomplishment. |
| **4** | **Managing Emotions** | Teaches users how to manage and regulate their emotions, particularly in stressful situations, by focusing on emotional awareness and healthy coping mechanisms |
| **5** | **Cognitive Restructuring** | Introduces users to the concept of challenging and reframing negative thoughts. It teaches how distorted thinking affects emotions and behaviors and how to replace such thoughts with more balanced perspectives. |
| **6** | **Problem Solving** | Emphasizes strategies for overcoming obstacles and making better decisions when faced with difficult situations, fostering a sense of control and agency. |
| **7** | **Healthy Relationships** | Helps users build and maintain healthy interpersonal relationships, focusing on communication skills, conflict resolution, and emotional support. |
| **8** | **Problem-Solving Difficult Situations** | Dives deeper into problem-solving techniques and the importance of perseverance in overcoming challenges, helping users develop resilience and confidence in their ability to handle adversity. |
| **9** | **The Social Network** | Aims to improve communication skills, foster strong social relationships, and resolve conflicts, emphasizing the role of social support in mental health and goal achievement. |
| **10** | **Communication Styles and Solutions** | Focuses on refining communication techniques, with an emphasis on calm, assertive communication to solve problems effectively. |

**Table S2**: Group CBT sessions content

| **Session** | **Title** | **Content Summary** |
| --- | --- | --- |
| **1** | **Setting the Foundation - Understanding Emotions and Goals** | Introduces adolescents to the program, emphasizing the importance of understanding emotions and setting personal goals. Covers the basics of depression, its symptoms, and its impact on thoughts, feelings, and actions. Establishes group rules and fosters a supportive environment. |
| **2** | **Building Bridges - Mastering Communication Basics** | Presents the fundamentals of effective communication, including active listening and expressing themselves constructively. Role-playing exercises help them practice these skills in real-life scenarios. |
| **3** | **Deepening Connections - Advanced Communication Strategies** | Establishes deeper understanding of communication techniques, focusing on overcoming misunderstandings and fostering meaningful relationships. Adolescents reflect on their own communication habits and practice improvement strategies. |
| **4** | **Tackling Challenges - Introduction to Problem-Solving** | Explores problem-solving as a tool for managing stress and conflicts. Participants learn to identify challenges, brainstorm solutions, and evaluate outcomes in a structured manner. |
| **5** | **Refining Solutions - Advanced Problem-Solving Skills** | Builds on the previous session by focusing on applying problem-solving techniques to complex issues. Adolescents engage in group exercises to simulate real-life problem-solving scenarios. |
| **6** | **Overcoming Setbacks - Coping Strategies for Adversity** | Focuses on helping adolescents develop strategies to handle setbacks and maintain progress. Techniques include reframing negative thoughts, seeking support, and reinforcing learned skills during challenging times. |
| **7** | **Taking Control - Strengthening Emotional Resilience** | Helps adolescents learn to identify triggers for negative emotions and practice techniques to manage stress and build resilience. Introduces relaxation exercises and strategies to reinforce positive behaviors. |
| **8** | **Moving Forward - Reflecting and Planning for the Future** | Focuses on consolidating the skills learned, reflecting on progress, and setting long-term goals for maintaining emotional well-being. Adolescents create personalized plans to continue practicing the techniques independently. |

**Table S3:** Post-intervention responses of participants to the Preferences for Intervention Scale

|  | **Definitely Acceptable** | | **Probably Acceptable** | | **Probably not Acceptable** | | **Definitely not Acceptable** | |
| --- | --- | --- | --- | --- | --- | --- | --- | --- |
|  | **N** | **%** | **N** | **%** | **N** | **%** | **N** | **%** |
| Talking with PCP about what you can do to prevent depression. | 30 | 28.8% | 35 | 33.7% | 26 | 25.0% | 13 | 12.5% |
| Talking with PCP & agreeing to set up a regular schedule. | 27 | 26.0% | 38 | 36.5% | 21 | 20.2% | 18 | 17.3% |
| Start an exercise program with thirty minutes of aerobic activity individually | 52 | 50.0% | 39 | 37.5% | 6 | 5.8% | 7 | 6.7% |
| Talking with PCP about your feelings and situation. | 30 | 28.8% | 31 | 29.8% | 24 | 23.1% | 19 | 18.2% |
| Completing a questionnaire on Internet | 14 | 13.9% | 29 | 28.7% | 31 | 30.7% | 27 | 26.7% |
| Seek one-on-one counseling from a mental health specialist | 25 | 24.8% | 37 | 35.5% | 22 | 21.8% | 17 | 16.3% |
| Start an exercise program with thirty minutes of aerobic activity with a group. | 39 | 37.9% | 36 | 35.0% | 16 | 15.5% | 12 | 11.7% |
| Completing a questionnaire with PCP | 19 | 18.6% | 25 | 24.5% | 30 | 29.4% | 28 | 27.5% |
| Talking with PCP and agreeing to complete an e-program | 40 | 40.0% | 21 | 21.0% | 17 | 17.0% | 22 | 22.0% |
| Wait and get over naturally | 16 | 15.8% | 33 | 32.7% | 29 | 28.7% | 23 | 22.8% |
| Participating in youth religious group | 45 | 44.6% | 27 | 26.7% | 15 | 14.9% | 14 | 13.9% |
| Use anti-depressant drugs | 5 | 5.2% | 11 | 11.3% | 19 | 19.6% | 62 | 63.9% |
| Seek group counseling with six or more patients | 12 | 11.8% | 19 | 18.6% | 26 | 25.5% | 45 | 44.1% |
| Take health class to learn about feelings and coping skills. | 24 | 23.5% | 43 | 42.2% | 22 | 21.6% | 13 | 12.7% |
| Talking to a counselor through phone about your feelings | 15 | 14.9% | 20 | 19.8% | 32 | 31.7% | 34 | 33.7% |
| Group meeting with 6-10 peers from your school. | 12 | 12.4% | 28 | 28.9% | 25 | 25.8% | 32 | 33.0% |

**Table S4:** Pre-intervention responses of participants to the Preferences for Intervention Scale

|  | **Definitely Acceptable** | | **Probably Acceptable** | | **Probably not Acceptable** | | **Definitely not Acceptable** | |
| --- | --- | --- | --- | --- | --- | --- | --- | --- |
|  | **N** | **%** | **N** | **%** | **N** | **%** | **N** | **%** |
| Talking with PCP about what you can do to prevent depression. | 12 | 12.4% | 26 | 26.8% | 26 | 26.8% | 33 | 34.0% |
| Talking with PCP & agreeing to set up a regular schedule. | 14 | 14.4% | 27 | 27.8% | 32 | 33.0% | 24 | 24.7% |
| Start an exercise program with thirty minutes of aerobic activity individually | 22 | 22.7% | 23 | 23.7% | 27 | 27.8% | 25 | 25.8% |
| Talking with PCP about your feelings and situation. | 14 | 15.2% | 27 | 29.3% | 25 | 27.2% | 26 | 28.3% |
| Completing a questionnaire on Internet | 16 | 16.8% | 24 | 25.3% | 28 | 29.5% | 27 | 28.4% |
| Seek one-on-one counseling from a mental health specialist | 12 | 12.6% | 22 | 23.2% | 24 | 25.3% | 37 | 38.9% |
| Start an exercise program with thirty minutes of aerobic activity with a group. | 19 | 19.6% | 29 | 29.9% | 25 | 25.8% | 24 | 24.7% |
| Completing a questionnaire with PCP | 13 | 14.0% | 19 | 20.4% | 31 | 33.3% | 30 | 32.3% |
| Talking with PCP and agreeing to complete an e-program | 18 | 18.9% | 22 | 23.2% | 31 | 32.6% | 24 | 25.3% |
| Wait and get over naturally | 13 | 13.4% | 30 | 30.9% | 25 | 25.8% | 29 | 29.9% |
| Participating in youth religious group | 20 | 21.3% | 19 | 20.2% | 27 | 28.7% | 28 | 29.8% |
| Use anti-depressant drugs | 11 | 12.0% | 25 | 27.2% | 26 | 28.3% | 30 | 32.6% |
| Seek group counseling with six or more patients | 13 | 13.4% | 22 | 22.7% | 31 | 32.0% | 31 | 32.0% |
| Take health class to learn about feelings and coping skills. | 15 | 16.0% | 33 | 35.1% | 19 | 20.2% | 27 | 28.7% |
| Talking to a counselor through phone about your feelings | 18 | 19.1% | 26 | 27.7% | 24 | 25.5% | 26 | 27.7% |
| Group meeting with 6-10 peers from your school. | 44 | 43.1% | 28 | 27.5% | 17 | 16.7% | 13 | 12.7% |
