# Supplementary figures and images for: Validating a digital depression prevention program for adolescents in Jordan: cultural adaptation and user testing in a randomized controlled trial
Source: Front Psychiatry. 2025 Feb 12;16:1529006. doi: 10.3389/fpsyt.2025.1529006 (PMC11860973; doi:10.3389/fpsyt.2025.1529006)

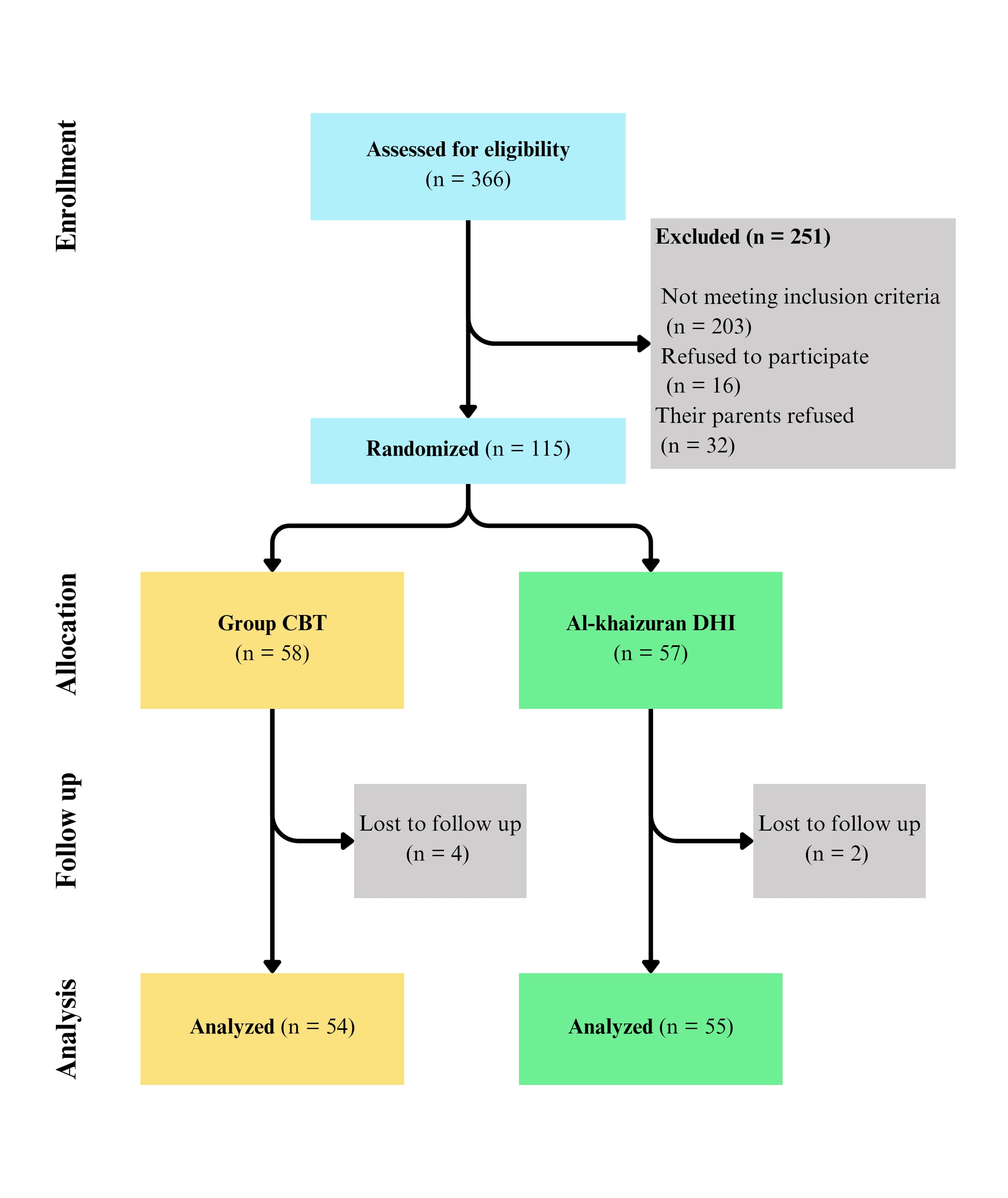

Supplement: Supplementary Figure 1 — CONSORT flow diagram. [file SupplementaryFile1.zip › SUPPLEMENTARY FIGURE 1 CONSORT flow diagram.jpeg]
